# The uses and abuses of Facebook: A review of Facebook addiction

**DOI:** 10.1556/JBA.3.2014.016

**Published:** 2014-08-26

**Authors:** TRACII RYAN, ANDREA CHESTER, JOHN REECE, SOPHIA XENOS

**Affiliations:** ^1^School of Health Sciences, RMIT University, Melbourne, Victoria, Australia; ^2^School of Design and Social Context, RMIT University, Melbourne, Victoria, Australia

**Keywords:** Facebook, social networking sites, addiction, uses and gratifications

## Abstract

*Background and aims:* Recent research suggests that use of social networking sites can be addictive for some individuals. Due to the link between motivations for media use and the development of addiction, this systematic review examines Facebook-related uses and gratifications research and Facebook addiction research. *Method:* Searches of three large academic databases revealed 24 studies examining the uses and gratifications of Facebook, and nine studies of Facebook addiction. *Results:* Comparison of uses and gratifications research reveals that the most popular mo- tives for Facebook use are relationship maintenance, passing time, entertainment, and companionship. These motivations may be related to Facebook addiction through use that is habitual, excessive, or motivated by a desire for mood alteration. Examination of Facebook addiction research indicates that Facebook use can become habitual or excessive, and some addicts use the site to escape from negative moods. However, examination of Facebook addic- tion measures highlights inconsistency in the field. *Discussion:* There is some evidence to support the argument that uses and gratifications of Facebook are linked with Facebook addiction. Furthermore, it appears as if the social skill model of addiction may explain Facebook addiction, but inconsistency in the measurement of this condition limits the ability to provide conclusive arguments. *Conclusions:* This paper recommends that further research be performed to establish the links between uses and gratifications and Facebook addiction. Furthermore, in order to enhance the construct validity of Facebook addiction, researchers should take a more systematic approach to assessment.

## Introduction

**Figure 1.**
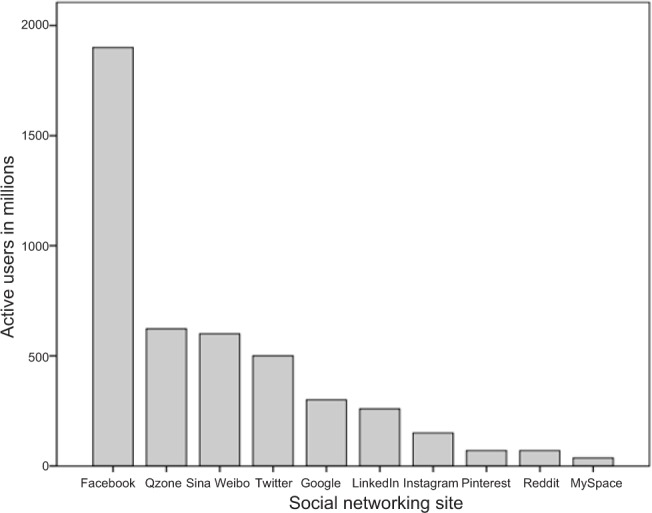
Active users of ten popular social networking sites

*Note:* Usage statistics in Figure 1 are current as at December 2013, and were sourced from the webpage “How Many People Use 340 of the Top Social Media, Apps & Services?” by Craig Smith, 1 December, 2013. Retrieved 9 December, 2013, from http://expandedramblings.com/index.php/resource-how-many-people-use-the-top-social-media.

In the last decade, the use of social networking sites (SNSs) has grown exponentially. For example, statistics provided by Facebook (2014) reveal that as of March 2014 there were 1.28 billion active users on the site per month, and at least 802 million of these users logged into Facebook every day. With statistics such as these, it is not surprising that Facebook is the most popular SNS in the world (see Figure 1). It is also one of the most popular websites on the Internet, second only to Google in global usage (Alexa Internet, 2013). As a result of this popularity, social scientists have recently begun to examine aspects of its use (for a detailed review of this topic see Wilson, Gosling & Graham, 2012). However, limited research has examined the potential for Facebook use to become addictive (Griffiths, Kuss & Demetrovics, 2014).

### SNS addiction

SNS addiction has been defined as a failure to regulate usage, which leads to negative personal outcomes (LaRose, Kim & Peng, 2010). While a growing number of researchers accept the possibility that the use of online applications can become addictive, the concept is contentious (Griffiths, 2013) . In fact, despite over 15 years of Internet addiction research, the most recent version of *The Diagnostic and Statistical Manual of Mental Disorders* (5^th^ ed.; *DSM-5;* American Psychiatric Association, 2013) failed to include it as an addictive disorder.

While the exclusion of Internet addiction from the *DSM-5* may create the perception that online addictions are not legitimate mental disorders, there is a large body of literature that suggests otherwise (see Kuss, Griffiths, Karila & Billieux, 2014, for a more extensive review of this topic).

In addition, a member of the *DSM-5* working group suggested that inclusion of Internet addiction in future iterations of the *DSM* is possible, but is contingent upon the results of more rigorous research studies (O’Brien, 2010). Unfortunately, at this point, there remains a sense of conceptual confusion associated with Internet addiction (Meerkerk, van den Eijnden, Vermulst & Garretsen, 2009). For instance, a recent systematic review identified that there is no gold standard measure of this condition, nor is there any widely accepted theory (Kuss et al., 2014).

One emerging theory of online addiction is Caplan’s (2010) social skill model of generalised problematic Internet use. This model states that individuals who prefer to communicate in an online environment are at greater risk of experiencing negative outcomes related to excessive online use. These individuals, who demonstrate *deficient self-regulation* of Internet use, tend to engage in online social communication as a means of escaping from negative mood states, such as loneliness or anxiety. Communicating online alleviates negative moods (known as *mood alteration*), which then reinforces online use. Given the social focus of SNSs, this theory has the potential to explain SNS addiction. However, despite the popularity of SNS use, empirical research examining addiction to these online social platforms is currently lacking.

In 2011, Kuss and Griffiths performed a comprehensive literature review to examine the legitimacy of SNS addiction. In their paper, they focused on six areas associated with SNS addiction: usage patterns, motivations for SNS use, personalities of SNS users, negative consequences of SNS use, empirical evidence of SNS addiction, and co-morbidity. At that time, the authors were only able to locate five studies of SNS addiction. As a result, they were limited in their ability to ascertain the status of this potential disorder. While they were able to recognise that excessive use of SNSs can be linked to negative outcomes, they concluded that more extensive research was required to prove the existence of this disorder.

Three years later, Griffiths et al. (2014) performed another review of SNS addiction, this time locating 17 studies. This increase in the extant literature highlights the perceived salience of this topic of investigation. However, despite the larger body of research available for review, Griffiths et al. were not able to offer any more substantial conclusions. While they did find preliminary evidence for some symptoms of SNS addiction (e.g., preoccupation, withdrawal, and negative consequences), methodological issues associated with the majority of studies precluded the ability to form any conclusions regarding the legitimacy of SNS addiction. As a result, they proposed that the question of whether addiction to SNSs exists remains open for debate.

Griffiths et al. (2014) also made the valid point that describing SNS addiction is not a clear-cut process. In particular, they posit that becoming addicted to the social aspects of SNS use may represent “cyber-relationship addiction” (Young, Pistner, O’Mara & Buchanan, 1999), while addiction to SNS games, such as the popular Facebook application *Farmville,* should fall under the classification of “gaming addiction” (Griffiths, 2012). In the present paper, we argue that this notion should be taken one step further; just as the Farmville addict may differ from someone who compulsively posts social content on SNSs, so too may the motivations of the Facebook addict differ from the Twitter addict. As will become clear, this point is supported by research relating to the gratifications of SNS use.

### Uses and gratifications of SNSs

Commonly, when researchers choose to examine the motivations associated with particular forms of media, they do so by employing a *uses and gratifications* approach. Uses and gratifications theory states that one of the keys to understanding the popularity of mass media lies in the identification of the factors underlying its use (Katz, Blumler & Gurevitch, 1973). One of the first studies to examine the uses and gratifications of SNSs was performed by Raacke and Bonds-Raacke (2008). After surveying a sample of university students from the USA, these authors reported that the primary motivations for Facebook and MySpace use was to form and maintain social connections. Since that time, numerous studies have reinforced the importance of relationship maintenance as a key reason for Facebook use (e.g., Joinson, 2008; Sheldon 2008, 2009; Valentine, 2012). Indeed, Kuss and Griffiths (2011) argue that relationship maintenance is the main motivator for all SNS use.

However, studies looking at the uses and gratifications of SNSs other than Facebook tend to indicate that Kuss and Griffiths’ (2011) argument may be somewhat misleading. For example, Dunne, Lawlor and Rowley (2010) report that one of the most important uses and gratifications for Bebo use among teenage girls was impression management. In addition, research relating to video and image sharing SNSs (such as YouTube and Pinterest) indicate that the use of these sites is primarily influenced by the need for self-expression and entertainment (Gülnar, Balcé & Çakér, 2010; Mull & Lee, 2014). Given the varied features of different SNSs, these findings are hardly surprising. As Chen (2011) notes, “multiple media compete for users’ attention”, and “active users select the medium that meets their needs” (p. 759).

The results of these studies show that, while it is true that all SNSs serve a similar purpose - to facilitate social interaction through the efficient dissemination of information to a desired audience - the specific features of each individual site are often varied (Boyd & Ellison, 2007). For this reason, it is unwise to assume that the results of a study that focuses on one particular SNS can be generalised to every SNS that is currently in existence (Panek, Nardis & Konrath, 2013). Furthermore, important differences in SNS usage might be undetectable when data from different sites are combined (Hargittai, 2008). Therefore, in the case of literature reviews such as those performed by Kuss and Griffiths (2011), it seems that the assumption of SNS homogeneity might be misguided. On the contrary, we argue that the need to separate out results from specific sites is crucial to understanding the development of SNS addiction.

### Uses and gratifications and SNS addiction

Earlier, the point was made that the gratifications of a Facebook addict may differ from those of a Twitter addict. This example highlights the need for SNS addiction researchers to consider the motivations behind the use of addictive SNS platforms. According to Papacharissi and Mendelson (2011), “online media serve as functional alternatives to interpersonal and mediated communication, providing options or complements for aspects of an individual’s environment that are not as fulfilling” (p. 214). In certain circumstances, Internet users may lose control over use that was originally motivated by “active consideration of the gratifications of online behaviour” (Song, LaRose, Eastin & Lin, 2004, p. 390).

While the relationship between uses and gratifications and SNS addiction was previously recognised by Kuss and Griffiths (2011), limited research has been performed in this area. One of the first empirical studies to examine the relationship between SNS addiction and uses and gratifications was performed by Wan (2009). She studied use of the campus-based SNS *Xiaonei.com* amongst a sample of 335 Chinese college students. The results revealed that Xiaonei.com addiction was significantly associated with the motives of socialisation and relationship building. Similarly, another study based on a Greek sample of 1971 adolescents (Floros & Siomos, 2013) found that the motivations of seeking friendship, relationship maintenance, and escapism, along with impulsive use of the Internet, predicted more frequent SNS participation.

While the two studies mentioned above support the notion that SNS use can be associated with a desire to socialise and form relationships online, findings from other studies indicate that this is not always the case. For example, Huang (2012) examined SNS use among 1549 adolescents, and found that entertainment gratifications were the strongest predictor of SNS addiction. In another study, Chen and Kim (2013) revealed that there was a positive relationship between SNS addiction and using SNSs for diversion and self-presentation. Of course, given that all of these studies (with the exclusion of Wan, 2009) measured aggregated SNS use, it is possible that these contrasting results reflect different types of SNSs used by each sample. If so, this would contribute to the argument that SNSs researchers should focus on specific sites rather than SNS use in general.

### Rationale and scope of this review

As outlined above, the development of SNS addiction is likely to be linked to the gratifications associated with use of the particular site. The aim of this paper was to clarify this relationship by synthesising literature relating to the motivations for SNS use and SNS addiction. In doing so, the present paper builds upon a previous review of SNS literature by Kuss and Griffith (2011). Based on the issues outlined above, we argue that this review is necessary for two main reasons. First, although only three years has passed since Kuss and Griffiths’ original review was conducted, Griffiths et al. (2014) recently demonstrated that the extant literature has grown substantially in this time period. Second, previous reviews of SNS addiction have failed to examine results from particular social networking sites in isolation. As argued above, this approach may have obscured important results relating to the particular motivations of SNS use and SNS addiction. In contrast, the present review expands on the previous work by focusing only on research related to a single SNS: Facebook.

There were two main reasons for selecting Facebook over other SNSs. First, Facebook is considerably more popular than other SNSs (see Figure 1). The widespread acceptance of Facebook suggests that there maybe unique factors associated with this SNS that are working to gratify the needs of a large number of Internet users. Second, in their review of SNS addiction, Griffith et al. (2014) demonstrated that empirical studies based on Facebook outweigh studies focusing on any other SNS.

The synthesis of literature provided in this review should not only clarify the findings related to Facebook addiction, but will also help to address questions regarding the particular motivations of Facebook users, and whether these motivations are linked to the development of Facebook addiction. Furthermore, by performing a review of Facebook addiction literature at such an early stage, inconsistencies with the conceptualisation and assessment of this disorder can be identified. Through this process, recommendations for future research can be made, which should hopefully fortify the construct validity of this potential condition. If this can be achieved, Facebook addiction research would avoid the conceptual confusion that has consistently plagued Internet addiction research.

## Methods

A literature search was performed using the academic databases *ProQuest* (including *PsycInfo), ScienceDirect,* and *Web of Science.* These databases were selected as they provide access to a large number of scientific peer-reviewed journal articles and theses from multiple disciplines. Two types of research studies were of interest in the current study: those relating to the uses and gratifications of Facebook, and those relating to Facebook addiction. Searches for uses and gratifications studies were performed using the terms ‘Facebook’, ‘social networking sites’, ‘social network sites’, ‘motivations’, and ‘uses and gratifications’. Searches for studies of Facebook addiction were performed using the terms ‘addiction’, ‘problematic’, ‘abuse’, ‘compulsive’, ‘excessive’, ‘social networking sites’, ‘social network sites’, and ‘Facebook’.

Uses and gratifications studies were included in the review if they measured the motivations of Facebook use in general; therefore, studies were excluded if they only focused on specific features of Facebook (i.e. a particular Facebook game). Furthermore, given that the present review was focused on the uses and gratifications of Facebook, rather than those of other SNSs, studies were excluded if they measured aggregated uses and gratifications for multiple SNSs (even if they included Facebook). According to LaRose, Mastro and Eastin (2001), “uses and gratifications researchers typically start with descriptions of common media uses, obtain ratings of the frequency or importance of those uses, and factor analyse the results to obtain gratification factors that are then correlated with media use” (p. 396). However, as this systematic review was interested in identifying all of the possible uses and gratifications of Facebook use, studies were included even if they had not reported evidence of factor analysis. In cases where factor analysis had been performed, the percentages of variance explained by each factor were recorded where available. This information was included in order to ascertain whether certain motivators of Facebook use are more important than others.

In regard to Facebook addiction literature, studies were excluded if they focused on addiction to SNSs in general (even if this included Facebook) and only provided combined results from these multiple sites in an aggregated format. As explained above, this criterion was necessary to ensure that results relating to other SNSs were excluded. For similar reasons, studies considering the role of Facebook use in relation to Internet addiction were also excluded.

## Results and Discussion

Within this section, the results of the literature searches are presented, followed by a review of the common findings identified within the extant literature. Uses and gratifications studies are discussed first, including a section dedicated to a discussion of the variables associated with particular uses and gratifications. This is followed by a review of Facebook addiction studies, including an examination of the various instruments that were used to measure this construct.

### Uses and gratifications

Twenty-four studies were identified that examined the uses and gratifications of Facebook and met the criteria identified above. For ease of comparison, the results of these studies are displayed in Table 1. When the uses and gratifications factors are compared, some clear patterns emerge. In 14 out of the 16 studies where the percentage of variance for each factor was reported, the factors accounting for the majority of the variance relate to either *relationship maintenance* or *passing time*. In this context, relationship maintenance involves interacting with members of an individual’s existing offline social network (Sheldon, 2008). Clearly, many Facebook users view the site as a useful tool to facilitate social interaction with existing friends and family. In this regard, Facebook differs from many older online social applications, such as discussion boards and newsgroups, which were primarily used for the formation of new relationships. Instead, Facebook appears to have an offline-to-online social focus (Ellison, Steinfield & Lampe, 2007).

Similar to the results presented here, Kuss and Griffiths (2011) also found that relationship maintenance was an important motivation for SNS use. As those authors did not look at specific SNSs independently from each other, it is unclear whether all SNSs have this focus, or whether these authors primarily discussed results from predominantly Facebook-related studies. The latter explanation is possible as, due to the popularity of the site, Facebook-related research tends to be more prominent than research relating to other SNSs. Clearly, researchers should endeavour to determine whether the uses and gratifications of other popular SNSs are similar or different to those associated with Facebook. In doing so, it would establish whether the popularity of Facebook is tied to unique factors.

In regard to the popular gratification of passing time, the findings appear to reflect the habitual use of Facebook to occupy time when bored, or to procrastinate from other activities (Foregger, 2008; Sheldon, 2008). Using Facebook for this purpose may involve such activities as checking the News Feed for new updates or playing games. Papacharissi and Mendelson (2011) refer to such use as ritualised, and indicate that it reflects “the addictive nature of the genre” (p. 226). Based on this, it is possible that the gratification of passing time may be related to Facebook addiction, but further research is required.

If the remaining factors in Table 1 are compared, it is apparent that *entertainment, companionship,* and *escape* appear across multiple studies. Although these factors tend to account for less variance in their respective analyses than relationship maintenance and passing time, they are also worth discussing briefly, as they may be related to the development of Facebook addiction.

Fifteen studies in Table 1 include a factor relating to the use of Facebook for *entertainment* purposes. This factor encapsulates using Facebook to engage in socially passive activities, such as looking at user-generated content on the site, or playing games. In essence, the entertainment factor appears similar in nature to the more popular passing time factor. However, the latter appears to be motivated more by task avoidance, procrastination or filling time, while the former reflects planned usage for the purposes of pleasure seeking. In Sheldon’s (2008) study, the entertainment factor had a high mean score, which highlights the importance of this motivation for Facebook use in certain populations.

In regard to *companionship,* this factor was present in six out of 24 studies. Companionship taps into the use of Facebook to avoid loneliness and gratify interpersonal needs. Similarly, two other studies included motivations that related to decreasing loneliness (Balakrishnan & Shamim, 2013; Teppers, Luyckx, Klimstra & Goossens, 2014). Given that there is a link between loneliness and the development of Internet addiction (Caplan, 2010), it is possible that factors such as these may also be related to Facebook addiction. It is interesting to note that in Valentine’s (2011) study, top-loading items in the companionship factor related to the use of Facebook to escape from worries and problems. Such items may be suggestive of mood alteration, which, as mentioned earlier, is linked to addiction of online social applications (Caplan, 2010; Lortie & Guitton, 2013). However, none of the uses and gratifications studies reviewed here explicitly referred to this dimension. Instead, they appear to use the term *escape*, which was included in four out of 24 studies.

**Table 1.** Systematic review of studies of the uses and gratifications of Facebook

Author(s)	Year	Sample	Motivations	Variance
				explained (%)
Foregger	2008	340 introductory communications	Pass time	32.6
		students (62% women) from	Connection	7.9
		Michigan State University, USA	Sexual attraction	5.0
			Utilities and upkeep	3.2
			Establish/maintain old ties	2.7
			Accumulation	2.7
			Social comparison	2.5
			Channel use	2.0
			Networking	1.8
Joinson^a^	2008	137 Facebook users (64% women),	Social connection	59^b^
		with a mean age of 26 years	Shared identities	
			Photographs	
			Content	
			Social investigation	
			Social network surfing	
			Status updating	
Sheldon	2008	172 communications students	Relationship maintenance	31
		(57% women) from Louisiana	Passing time	11.2
		State University, USA	Virtual community	5.2
			Entertainment	4.6
			Coolness	4.2
			Companionship	4
Sheldon	2009	260 communications students	Relationship maintenance	31.1
		(58% women) from Louisiana	Passing time	9.7
		State University, USA	Entertainment	4.8
			Virtual community	4.1
Cheung, Chiu & Lee	2011	182 Facebook users (68% women,	Social presence	Not reported
		86% students)	Entertainment value	
			Social enhancement	
			Group norms	
			Maintaining interpersonal interconnectivity	
Hart	2011	163 final year high school students	Passing time	29.3
		(57% women) from USA	Relationship maintenance	10.4
			Entertainment	7.5
			Information seeking	5.3
Hart	2011	186 undergraduate university	Relationship maintenance	38.4
		students (65% women) from USA	Passing time	9.3
			Entertainment	7.2
			Information seeking	4.8
Papacharissi &	2011	344 Facebook users (64.3% women).	Habitual pass time	11.4
Mendelson		85% were undergraduate university	Relaxing entertainment	10.5
		students from USA	Expressive information sharing	9.4
			Cool and new trend	7
			Companionship	6.8
			Professional advancement	6.7
			Escape	6.6
Smock, Ellison,	2011	267 undergraduate communications	Social interaction	Not reported
Lampe & Wohn		students (65% men) from a large	Habitual pass time	
		midwestern USA university	Relaxing entertainment	
			Expressive information sharing	
			Escapism	
			Cool and new trend	
			Companionship	
			To meet new people	
			Professional advancement	
Alhabash, Park,	2012	4,346 Taiwanese Facebook users	Social connection	5.87
Kononova, Chiang		(59% women) with a mean age	Photographs	3.48
& Wise		of 30 years	Social investigation	3.36
			Status updates	2.72
			Social network surfing	2.70
			Content	2.50
			Shared identities	2.44
Hunt, Atkin	2012	417 undergraduate students. No	Interpersonal utility	Not reported
& Krishnan		further demographic information	Passing time	
		about participants was provided	Information seeking	
			Entertainment	
			Self-expression	
Special & Li-Barber	2012	127 undergraduate Psychology	Relationship maintenance	Not reported
		students (71% women) from a	Passing time	
		small southeastern USA university	Entertainment	
			Coolness	
			Virtual community	
			Companionship	
Tosun	2012	143 Turkish university students	Managing long-distance relationships	12.98
		(74% women)	Passive activities	11.84
			Initiating/terminating romantic relationships	10.23
			Establishing new relationships	10.14
			Active forms of photo-related activities	8.25
			Game/entertainment	7.67
			Organising events	7.42
Valentine	2012	350 Internet users (69% women)	Interpersonal habitual entertainment	37.54
		from the USA. All were over	Virtual companionship	9.65
		35 years of age	Information seeking	5.73
			Self expression	3.91
			Passing time	3.45
Yang & Brown	2012	193 university students (54%	Relationship formation	0.30
		women) from a large midwestern	Relationship maintenance	0.10
		USA university		
Balakrishnan	2013	707 university students from	Social networking	35.10
& Shamim		Malaysia (54% women)	Psychological benefits	10.5
			Entertainment	8.72
			Self presentation	5.26
			Skill enhancement	3.32
Giannakos,	2013	222 Facebook users (56% men),	Wasting time	32.20
Chorianopoulos,		with a mean age of 26 years	Social connection	14.54
Giotopoulos			Social surfing	13.42
& Vlamos			Using applications	9.24
Pai & Arnott	2013	24 Taiwanese Facebook users	Belonging	Not reported
		(50% women) aged 20-40 years	Hedonism	
			Self-esteem	
			Reciprocity	
Spiliotopoulos	2013	208 Facebook users (44.2%	Social connection	69.01^b^
& Oakley^a^		women) from 30 different countries	Shared identities	
			Photographs	
			Content	
			Social investigation	
			Social network surfing	
			Newsfeed	
Teppers, Luyckx,	2014	256 senior high school students	Entertainment	Not reported
Klimstra & Goossens		(64% girls) from Belgium	Maintaining relationships	
			Social skills compensation	
			Social inclusion	
			Meeting people	
			Decrease loneliness	
			Personal contact	
Aladwani	2014	378 student Facebook users	Connecting	24.00
		from a university in Kuwait	Sharing	10.40
		(55% men)	Organising	8.18
			Branding	7.11
			Expressing	6.82
			Monitoring	6.70
			Learning	6.37
			Relaxing	5.80
Alhabash, Chiang	2014	3172 Taiwanese Facebook users	Information sharing	78.99
& Huang		(50% women)	Self-expression	74.83
			Self-documentation	73.61
			Medium appeal	70.57
			Socialisation	70.05
			Entertainment	61.90
			Escapism	54.16
Hollenbaugh & Ferris	2014	301 Facebook users (77% women),	Virtual community	18.13
		with a mean age of 31.85 years	Companionship	17.45
			Exhibitionism	14.68
			Relationship maintenance	14.63
			Passing time	6.71
Shoenberger	2014	123 students from a large	Affectation	26.00
& Tandoc, Jr		midwestern USA university	Bandwagon	14.83
			Self-expression	11.18
			Entertainment	7.09
			Escape	6.05
			Companionship	5.04
			Excitement	4.44
			Sociability	3.40

^a^As these results originate from conference papers, they may be of a lower quality than the other reported studies.

^b^Individual variances for each factor were not provided.

### Variables linked to uses and gratifications

Several of the 24 studies in Table 1 also identified variables that are commonly linked to the uses and gratifications of Facebook. A discussion of these variables was deemed to be germane to the current paper, as it sheds light on the types of people who may be at risk of Facebook addiction. This discussion taps into three main variables: gender, frequency of use, and duration of use.

Of the studies presented in Table 1, five examined the association between gender and uses and gratifications of Facebook (Hunt, Atkin & Krishnan, 2012; Joinson, 2008; Sheldon, 2009; Spiliotopoulos & Oakley, 2013; Teppers et al., 2014). In all of these studies, women were more likely than men to use Facebook for connecting with existing contacts. In contrast, Sheldon (2009) found that men were more likely than women to be motivated by making new friends or forming new romantic relationships on Facebook. Although Facebook has changed since Sheldon’s study was published, a recent study by Spiliotopoulos and Oakley (2013) also found that men prefer to use Facebook to engage in social network browsing.

The above results point to a fundamental difference between women and men in their uses and gratifications of Facebook; women prefer to use the site to maintain their existing social networks, while men prefer to use it to expand their social networks. Given that past research has linked Internet addiction with a tendency to prefer communicating with new online friends (e.g., Morahan-Martin & Schumacher, 2000; Young, 1998a), it is possible that men may be more likely to fail to regulate their online communication and become addicted to Facebook. However, recent research has found that women are heavier users of Facebook than men (Foregger, 2008). In light of these conflicting results, it is clear that researchers should examine the difference that gender plays in the development of Facebook addiction. In fact, it may be the case that there are multiple pathways to addiction, and these are mediated by different communicative motivations.

In Joinson’s (2008) study, frequency of Facebook use - that is, returning to Facebook multiple times per day - was found to be associated with what he called *surveillance gratifications*. This involves looking at user-generated content, such as photographs and status updates. Similarly, Hart (2011) reported that the entertainment gratification was a significant variable in a model predicting the frequency of Facebook use in both undergraduate and high school students. These results imply that passively engaging with social or entertainment-related content on Facebook can motivate users to return to the site frequently. This kind of use may be associated with checking for real-time updates on the News Feed, as content will generally be updated regularly. Such behaviour may be tapping into what is anecdotally referred to as *fear of missing out* or *FoMO* (Przybylski, Murayama, DeHaan & Gladwell, 2013); however, this warrants further investigation.

Interestingly, Papacharassi and Mendelson (2011) found that people who used Facebook more frequently developed a greater affinity with the site, especially when they used it to escape from negative emotions. As already discussed, the use of online applications for mood alteration is associated with deficient self-regulation and negative outcomes (Caplan, 2010). Therefore, it is possible that this aspect of the social skill model of generalised problematic Internet use is relevant to the use of Facebook. While more in-depth research is required to support this theory, it is plausible that lonely or socially anxious individuals may feel more connected with others when checking the News Feed for recent updates, or when receiving messages or comments from friends. If so, this may lead such users to check the site frequently, in order to attain the negative reinforcement of mood alteration.

Joinson (2008) also found that the duration of time spent on Facebook per day was predicted by what he referred to as *content gratifications,* which involve engaging in non-so- cially oriented Facebook activities (i.e., playing games, searching applications, and completing quizzes). Similarly, Foregger (2008) found that using Facebook to pass time led to more time spent on Facebook per day. Taken together, these findings suggest that individuals who spend a lot of time on Facebook per day may do so for different reasons than those who check Facebook frequently. For example, rather than passively engaging with posted social content in the way that frequent users do, heavy users may be gratified by non-social activities such as game playing.

In contrast to the assumption above, Hart (2011) discovered that using Facebook for entertainment and relationship maintenance significantly contributed to a model predicting the amount of time spent on Facebook per day. This opposing result can potentially be explained by changes made to Facebook after 2008. In particular, Facebook added the real-time synchronous instant messaging application ‘Chat’ in April of that year (Wiseman, 2008). This feature may have encouraged some Facebook users to spend more time on the site for social purposes, such as chatting with their friends and family. Furthermore, Alhabash, Park, Kononova, Chiang and Wise (2012) reported that Facebook intensity was predicted by the desire to share personal information via status updates. These results suggest that socially active Facebook applications, such as Chat and status updates may be associated with heavy Facebook use. One potential explanation for this trend is that the use of these applications increases the chance of receiving comments and messages from other users. For some individuals, such as those who are lonely, receiving this type of feedback could provide relief from feelings of social isolation and reinforce the use of these applications. In support of this, Yang and Brown (2013) reported that the use of status updates was associated with higher levels of loneliness, while Teppers et al. (2014) found that lonely adolescents were more likely to use the socially interactive applications of Facebook than non-lonely adolescents.

### Facebook addiction

Nine studies measuring Facebook addiction were located through the literature searches (see Table 2). The results of these studies suggest that Facebook addiction is associated with being male (Çam & İsbulan, 2012), being a heavy Facebook user (Hong, Huang, Lin & Chiu, 2014; Koc & Gulyagci, 2013), and being in a higher year level at university (Çam & İsbulan, 2012). Facebook addiction was also linked to certain psychological variables, such as relationship dissatisfaction (Elphinston & Noller, 2011), depression (Hong et al., 2014; Koc & Gulyagci, 2013), anxiety (Koc & Gulyagci, 2013), subjective happiness, and subjective vitality (Uysal, Satici & Akin, 2014). In terms of the symptoms of Facebook addiction, support was found for the existence of preference for online social interaction, mood alteration, deficient self-regulation, negative outcomes (Lee, Cheung & Thadani, 2012), salience, loss of control, withdrawal, relapse (Balakrishinan & Shamim, 2013), and tolerance (Zaremohzzabieh, Samah, Omar, Bolong & Kamarudin, 2014).

**Table 2.** Systematic review of Facebook addiction studies

Author(s)	Year	Sample	Type of study	Measure	Variables	Findings
Elphinston & Noller	2011	342 Australian undergraduate students (57% women)	Quantitative cross-sectional survey study	8-item Facebook Intrusion Questionnaire	Facebook intrusion, jealousy, relationship satisfaction	Facebook intrusion is associated with relationship dissatisfaction through jealousy and surveillance behaviours
Sofiah, Omar, Bolong & Osman	2011	380 Malaysian university students (100% women)	Quantitative cross-sectional survey study	11-item unnamed measure of Facebook addiction	Facebook addiction, uses and gratifications of Facebook	Social interaction, passing time, entertainment, companionship and communication motives were all associated with Facebook addiction
Çam & Isbulan	2012	1257 teaching candidates from a Turkish university (59% women)	Quantitative cross-sectional survey study	20-item Facebook Addiction Scale	Facebook addiction, gender, year of study	Men were more likely than women to be addicted to Facebook, and senior students were more likely to be addicted than juniors, sophomores, and freshmen
Lee, Cheung & Thadani^a^	2012	200 Facebook users (52% women)	Quantitative cross-sectional survey study	7-item modified version of the Generalised Problematic Internet Use Scale 2 (Caplan, 2010)	Problematic Facebook use	Preference for online social interaction and using Facebook to regulate moods significantly predicted deficient self-regulation of Facebook use. This relationship led to negative outcomes
Balakrishinan & Shamim	2013	Focus group: 12 Malaysian university students Survey: 707 Malaysian university students (54% women)	Qualitative focus group study/Quantitative cross-sectional survey study	30-item unnamed measure of Facebook addiction	Facebook addiction, uses and gratifications of Facebook	Evidence was presented to support four key indicators of Facebook addiction: Salience, Loss of Control, Withdrawal, Relapse and Reinstatement
Koc & Gulyagci	2013	447 Turkish university students	Quantitative cross-sectional survey study	8-item Facebook Addiction Scale	Facebook addiction, Facebook use, psychosocial health	22% of the variance in Internet addiction scores was predicted by weekly time spent on Facebook, social motives, depression and anxiety
Hong, Huang, Lin & Chiu	2014	241 Taiwanese university students (59% men)	Quantitative cross-sectional survey study	12-item Facebook Addiction Scale	Facebook addiction, Facebook usage, gender, year of study, self-esteem, social extraversion, sense of self-inferiority, neuroticism, depressive character	Facebook addiction was significantly predicted by level of Facebook usage and having a depressive character
Uysal, Satici & Akin	2014	297 Turkish university students (53% women)	Quantitative cross-sectional survey study	18-item Bergen Facebook Addiction Scale (Andreassen, Torsheim, Brunborg & Pallesen, 2012)	Facebook addiction, subjective vitality, subjective happiness	The relationship between subjective vitality and subjective happiness was partially mediated by Facebook addiction
Zaremohzzabieh, Samah, Omar, Bolong, & Kamarudin	2014	9 heavy Facebook users from a Malaysian university (67% men)	Qualitative interview study	Semi-structured interview questions	Facebook addiction	Three themes emerged: compulsion to check Facebook, high frequency Facebook use, and using Facebook to avoid offline responsibility. These themes were respectively classified as salience, tolerance, and conflict

^a^As these results originate from a conference paper, they may be of a lower quality than the other reported studies.

Only one study directly examined whether there was an association between the uses and gratifications of Facebook and Facebook addiction. Sofiah, Omar, Bolong and Osman (2011) reported that Facebook addicts were more inclined to use Facebook for social interaction, passing time, entertainment, companionship, and communication. These findings support the assumptions made earlier following the systematic review of uses and gratifications studies. Despite the lack of direct examination of the motivations of Facebook use by addictions researchers, the results of the studies included in Table 2 tap into three distinct themes that were also apparent in the uses and gratifications research: habitual Facebook use, excessive Facebook use, and mood alteration. The following section will discuss these results in more detail. Following this, a discussion relating to the measurement of Facebook addiction in these studies will be provided.

### Habitual Facebook use

In the study performed by Elphinston and Noller (2011), the three items on the Facebook Intrusion Scale with the highest individual mean scores were ‘I often use Facebook for no particular reason’, ‘I feel connected to others when I use Facebook’, and ‘I lose track of how much I am using Facebook’. Likewise, Sofiah et al. (2011) reported that the items with the highest mean scores on their measure of Facebook addiction were ‘Facebook has become part of my daily routine’, ‘I find that I stay on Facebook longer than intended’, and ‘I feel out of touch when I haven’t logged onto Facebook for a while’. These results highlight the propensity for Facebook use to lead to deficient self-regulation through habitual and unmonitored use.

The results of the study by Sofiah et al. (2011) also revealed that the gratification of using Facebook to pass time accounted for 17.3% of the variance in scores from their measure of Facebook addiction (described in Table 3). Further, using Facebook for the combined motives of passing time, entertainment, and communication accounted for 23.9% of the variance. Therefore, habitual use of Facebook for passing time may put users at risk of Facebook addiction through the development of deficient self-regulation. As discussed above, passing time on Facebook appears to be predominantly associated with task avoidance and procrastination (Foregger, 2008; Sheldon, 2008). As these types of gratifications are not socially focused, it seems that Caplan’s (2010) social skill model may not be adequate to explain these particular results. Further research is warranted to explore this supposition.

### Excessive Facebook use

Two of the studies listed in Table 2 reported that higher levels of Facebook use were linked to Facebook addiction (Hong et al., 2014; Koc & Gulyagci, 2013). These results are not surprising, given that online addictions researchers have previously pointed to a link between heavy Internet usage and addiction (e.g., Tonioni et al., 2012). In fact, many scholars have used the term “excessive Internet use” interchangeably with the term Internet addiction. This trend is most likely due to the popular belief that spending a large amount of time performing a particular behaviour, such as exercise or eating chocolate, is an indicator of the presence of addiction (Leon & Rotunda, 2000); however, there are mixed views on this argument. Both Caplan (2005) and Griffiths (1999) have pointed out that excessive time spent online does not automatically qualify an individual as addicted. There are many non-problematic Internet behaviours that would involve extended periods of time online, such as study or work-related research. However, while not all people who spend large amounts of time on Facebook per day are necessarily addicted, due to the role that deficient self-regulation is thought to play, it makes sense that Facebook addicts would generally be heavy users.

Research relating to the uses and gratifications of Facebook has indicated that time spent on Facebook per day is related to content gratifications (Joinson, 2008), passing time (Foregger, 2008), and relationship maintenance (Hart, 2011). Frequency of Facebook use has also found to be associated with using Facebook for entertainment (Hart, 2011) and surveillance gratifications (Joinson, 2008). This suggests that there are several different gratifications associated with both heavy and frequent Facebook use, and again, not all are socially focused.

### Mood alteration

Lee et al. (2012) assessed whether Caplan’s (2010) social skill model applied to Facebook addiction. The results revealed that having a preference for online social interaction, and using Facebook for mood alteration, explained 35% of the variance in scores measuring deficient self-regulation of Facebook use. In turn, deficient self-regulation of Facebook use had a direct impact on the experience of negative life outcomes. While not measuring mood alteration directly, two other studies (Hong et al., 2014; Koc & Gulyagci, 2013) provided evidence to support a relationship between low psychosocial health (depression and anxiety) and Facebook addiction. These findings may indicate that depressed and anxious people turn to Facebook to find relief and escape.

In regard to the link between these findings and uses and gratifications, evidence suggests that lonely people use Facebook to gain a sense of companionship (Foregger, 2008; Sheldon, 2008), and to help them escape from their worries and problems (Valentine, 2012). Papacharassi and Mendelson (2011) found that people who use Facebook to escape from unwanted moods use the site more frequently. They also tend to enjoy Facebook use more than non-lonely users. In 2007, Caplan reported that loneliness is associated with Internet addiction, and that this relationship is mediated by social anxiety. Therefore, it seems that the findings reported here partly support Caplan’s (2010) social skill model.

### Measuring Facebook addiction

Due to the fact that Facebook addiction is an emerging field, different researchers have taken varying approaches to the measurement of this potential disorder. This is illustrated in Table 3, which provides a summary of existing Facebook addiction instruments. As can be seen, scholars have tended to either create their own measures based on research from related addiction fields, or they have borrowed and modified existing measures of Internet addiction. A similar process also occurred when researchers began to create measures of Internet addiction (Lortie & Guitton, 2013). Most Internet addiction instruments seem to be based on other addictive disorders, such as pathological gambling or substance-related addiction. This approach has led to confusion surrounding the appropriate criteria with which to measure Internet addiction, and has contributed to the underlying sense of conceptual chaos in the field (Meerkerk et al., 2009). As a result, applying a similar approach to the measurement of Facebook addiction should be avoided.

In support of the above argument, examination of the Facebook addiction instruments that have been subjected to factor analysis (see Table 3) highlights inconsistency in measurement. For instance, both The Facebook Intrusion

**Table 3.** Facebook Addiction Assessment Instruments

Elphinston & Noller	2011	Facebook Intrusion Questionnaire	Mobile phone involvement questionnaire and Brown’s (1997) behavioural addictions criteria	I often think about Facebook when I’m not using it I often use Facebook for no particular reason Arguments have arisen with others because of my Facebook use I interrupt whatever else I am doing when I feel the need to access Facebook I feel connected to others when I use Facebook I lose track of how much I am using Facebook The thought of not being able to access Facebook makes me feel distressed I have been unable to reduce my Facebook use	7-point scale	None provided
Sofiah, Omar, Bolong & Osman	2011	Untitled	Not reported	Facebook has become part of my daily routine I find that I stay on Facebook longer than I intended I feel out of touch when I haven’t logged onto Facebook for a while I think life without Facebook would be boring I tend to spend more time in Facebook over going out with others I often spent time playing games with friends through Facebook I often think about Facebook when I am not using it I often lose sleep due to late-night logins to Facebook I neglect everyday responsibilities to spend more time on Facebook My priority is to log on to Facebook rather than doing other things My grades are getting lower because of the amount of time I spend on Facebook	7-point scale	None provided
Andreassen, Torsheim, Brunborg & Pallesen	2012	Bergen Facebook Addiction Scale^ab^	Criteria of behavioural addiction (based on pathological gambling research). Wording was based on scale of gaming addiction.	How often during the past year have you: Spent a lot of time thinking about Facebook or planned use of Facebook? (Salience) Felt an urge to use Facebook more and more? (Tolerance) Use Facebook in order to forget about personal problems (Mood modification) Tried to cut down on the use of Facebook without success? (Relapse) Became restless or troubled if you have been prohibited from using Facebook? (Withdrawal) Use Facebook so much that it has had a negative impact on your job/studies? (Conflict)	5-point scale	None provided
Çam & Isbulan	2012	Facebook Addiction Scale	Modified version of Young’s (1998b) Internet Addiction Test	How often do you: Stay on Facebook longer than intended Neglect household chores to spend more time on Facebook Prefer the excitement of Facebook to intimacy with a partner Form new relationships with fellow Facebook users Hear others complain about the amount of time you spend on Facebook Grades or school-work suffers because of time spent on Facebook Check Facebook messages before something else that needs to be done Job performance or productivity suffers because of Facebook Become defensive or secretive when asked about Facebook activity Block out disturbing thoughts about your life with soothing thoughts of Facebook Find yourself anticipating when you will go on Facebook again Fear that life without Facebook would be boring, empty, and joyless	6-point scale	None provided
Çam & Isbulan (cont.)				Snap, yell, and act annoyed if someone bothers you while you are on Facebook Lose sleep due to late night Facebook logins Feel preoccupied with Facebook when offline, or fantasise about being on Facebook Say to yourself ‘just a few more minutes’ when on Facebook Try to cut down the amount of time spent on Facebook and fail Try to hide how long you’ve been on Facebook Choose to spend more time on Facebook over going out with others Feel depressed, moody, or nervous when offline, and having this feeling go away once back on Facebook		
Lee, Cheung & Thadani	2012	GPIUS2^a^	Modified version of the Generalised Problematic Internet Use Scale 2 (Caplan, 2010)	I want to, or have made unsuccessful efforts to, cut down or control my Facebook use (Deficient self-regulation) I have attempted to spend less time on Facebook but have not been able to (Deficient self-regulation) I have tried to stop using Facebook for long periods of time (Deficient self-regulation) I am preoccupied with Facebook if I cannot log on for some time (Deficient self-regulation) When not on Facebook, I wonder what is happening on there (Deficient self-regulation) I feel lost if I can’t go on Facebook (Deficient self-regulation) I have used Facebook to talk with others when I was feeling isolated (Mood regulation)	5-point scale	None provided
Balakrishnan & Shamim	2013	No title provided^2^	Brown’s (1997) behavioural addiction criteria	I spent a lot of time on Facebook (Salience) I might log into Facebook at least once daily (Salience) I constantly check for updates (Salience) Most of the time I spend on the Internet is for Facebook (Salience) I always reply to comments by my friends (Salience) Facebook has become part of life (Salience) I have the constant urge to update my status on Facebook (Salience) I go through my own profile regularly reading all the older posts (Salience) I use Facebook to check on people I met offline (Salience) I would be lost without Facebook (Salience) I think of Facebook when I am offline (Salience) Sometimes I think of Facebook while in my lecture/meeting/discussion (Salience) I think Facebook is the greatest invention ever (Salience) I lose sleep at times due to late night log-ins to Facebook (Loss of Control) I feel lost when I didn’t use Facebook for sometime (Loss of Control) I do not think I can stop using Facebook (Loss of Control) Facebook is affecting my offline life (academic, social life, etc.) (Loss of Control) I check every comment, photo, or video uploaded on my Facebook (Loss of Control) I am always online on Facebook so as not to miss any updates (Loss of Control) Sometimes I access the Internet just to get on Facebook (Loss of Control)	5-point scale	None provided
Balakrishnan & Shamim (cont.)				I lose track of time when I am on Facebook (Loss of Control) I get annoyed when someone disturbs me when I am using Facebook (Loss of Control) I get disappointed when I could not access Facebook (Withdrawal) I get disappointed when my friends are not online (Withdrawal) I get disappointed when my friend request is rejected (Withdrawal) I have deactivated my account before but I have activated it again (Withdrawal) I always look forward to using Facebook Others have commented that I spend too much time on Facebook Using Facebook is affecting my studies/work I have cancelled appointments before just to spend more time on Facebook		
Koc & Gulyagci	2013	Facebook Addiction Scale	Previous research on Internet addiction	I have difficulties in focusing on my academic work due to my Facebook use The first thing on my mind when I get up is to log into Facebook I lose sleep over spending more time on Facebook My Facebook use interferes with doing social activities I log into Facebook to make myself feel better when I am down My family or friends think that I spend too much time on Facebook I feel anxious if I cannot access Facebook I have attempted to spend less time on Facebook but have not succeeded	5-point scale	None provided
Hong, Huang, Lin & Chin	2014	Facebook Addiction Scale^a^	Modified version of Young’s (1998b) Internet Addiction Test	When you are not on Facebook, you will feel sad, in low spirits, and anxious, but after going on Facebook, these feelings will disappear (Withdrawal) When you are not on Facebook, will you still think about being on Facebook or imagine that you are on Facebook (Withdrawal) You would rather spend more time on Facebook than go out to spend time with people (Withdrawal) The time I spent on Facebook usually exceeds what I expected (Tolerance) I will overlook academic work to spend time on Facebook (Tolerance) Before I have to do something, I will check my Facebook to see if there is new information or there are games to play (Tolerance) When people ask me what I do on Facebook, I will become more defensive or private (Life problems) Because I spend too much time on Facebook, my academic work or grades have been affected (Life problems) My academic performance and attention have been affected by Facebook (Life problems) I like to make new friends on Facebook (Substitute satisfaction) I have discovered that I want to be on Facebook again (Substitute satisfaction) I am scared that without Facebook, life will become boring, empty, and uninteresting (Substitute satisfaction)	6-point scale	None provided

^a^These measures have been subjected to factor analysis.

^b^This paper was not included in Table 2 as it is an instrument development study rather than a Facebook addiction study.

Questionnaire (FIQ; Elphinston & Noller, 2011) and the Bergen Facebook Addiction Scale (BFAS; Andreassen, Torsheim, Brunborg & Pallesen, 2012) include factors tapping into salience, withdrawal and relapse; however, that is where the similarities between these measures end. Likewise, there are more differences than similarities between the Generalised Problematic Internet Use Scale (GPIUS2; Caplan, 2010) and the BFAS, although both include a mood-related factor (mood alteration/mood modification) and a negative outcomes factor (negative outcomes/conflicts). These examples underscore a lack of construct validity surrounding Facebook addiction. Moreover, they highlight the inconsistencies underlying behavioural addictions research in general.

As Facebook is an application of the Internet, it could be argued that the manifestation of Facebook addiction would have more in common with Internet addiction than it does with other forms of addiction, such as pathological gambling. In support of this claim, Caplan (2010) argues that preference for online communication is the key factor associated with the development of problematic use of online forms of communication. Given that Lee et al. (2012) found this factor was also relevant to Facebook addiction, it seems that preference for online social interaction is a factor worth including in ameasure of Facebook addiction. The modified version of the GPIUS2 therefore possibly presents the best option for measuring Facebook addiction out of all of the measures in Table 3; however, it also has limitations. For example, it does not provide a cut-off point for recognising problematic use (Spraggins, 2009), nor does it measure how long the use has been problematic (Griffiths, 2000).

Another point to consider is that, in light of the unprecedented popularity of Facebook with Internet users across the world, it is possible that there may be unique aspects associated with the development of addiction to this site. For example, past research has linked Internet addiction to the desire to communication with new online acquaintances, but uses and gratifications research has shown that the main motivation of Facebook use relates to maintaining existing online relationships. In this way, Facebook may be different to other forms of social media; however, this has yet to be determined.

Furthermore, if it is true that maintaining existing online relationships leads to Facebook addiction, it is important to be clear about what ‘existing relationships’ means. Does it refer purely to current and strong existing offline relationships, or does it take into account relationships from the past that have been rekindled through Facebook? One way of answering such questions would be to conduct in-depth exploratory research with Facebook addicted individuals. As opposed to borrowing and amending measures from conceptually related disorders, proceeding with research in an exploratory direction could enhance the construct validity of Facebook addiction and its associated measures.

## Conclusions

The aim of this paper was to extend the work of Kuss and Griffiths (2011) by synthesising literature relating to the uses and abuses of Facebook. By examining this research, several important and previously unreported points have been highlighted. First, researchers have recognised that the main uses and gratifications of Facebook are relationship maintenance, passing time, entertainment, and companionship. Some of these gratifications appear to be more common among particular groups, such as women and younger users. Although there is limited empirical research examining the links between uses and gratifications and Facebook abuse, it is possible that these motives may cause Facebook use that is habitual, excessive, or motivated by a desire to escape from negative moods.

Second, in regard to Facebook addiction, the findings discussed here paint the following picture: individuals with low psychosocial wellbeing, such as loneliness, anxiety or depression, are motivated to use Facebook to find social support or to pass time. The lift in mood that this provides (also known as mood alteration) leads to deficient self-regulation, possibly due to negative reinforcement. In severe cases, this can eventually lead to negative life consequences.

For the most part, this description appears to support Caplan’s (2010) social skill model of generalised problematic Internet use. On the other hand, it is also possible that there are multiple pathways to Facebook addiction; for instance, those triggered by non-socially motivated use or fear of missing out. Unfortunately, at this point in time, inconsistency in the measurement of Facebook addiction makes it difficult to propose compelling arguments regarding this condition. It seems, therefore, that researchers should focus on strengthening the assessment of Facebook addiction before examining alternative pathways to the development of this condition. Further research should also aim to explore Facebook use within the general population, rather than focusing primarily on university students.

Furthermore, the offline-to-online social interactions that appear to motivate most Facebook users may be different to other forms of social media. Therefore, when measuring Facebook addiction, it is important to use an instrument that takes into account the potentially unique symptoms of the condition. At present, the existing measures described within this paper fail to achieve this, as they are primarily based on research from other areas of addiction. While the inclusion of the core symptoms of addiction is important, researchers in this area should also aim to conduct detailed exploratory studies of Facebook addiction, using either qualitative or mixed methods. This process should facilitate the development of more focused instruments of Facebook addiction, which, in turn, should provide more concrete evidence to support the legitimacy of this addictive disorder.

### Limitations

Prior to concluding this paper, it is worth mentioning the possibility that performing a meta-analysis rather than a systematic review may have led to greater understanding of the uses and gratifications of Facebook and Facebook addiction. It should be mentioned, however, that a lack of consistency in regard to Facebook addiction measurement made a metaanalytic approach difficult.

### Broader implications

It appears as if there is some evidence to support the notion that the uses and abuses of Facebook are linked. At this point in time, however, research addressing this salient area is still in its infancy. While some tentative steps forward have been made with this review, it is clear that the construct validity of Facebook addiction and its associated measures must be strengthened before research continues.

In addition, there is a strong need for a systematic method of item development when measuring emerging forms of addictive behaviours. As demonstrated in the present review, researchers currently tend to take a haphazard approach, which could end up resulting in conceptual confusion. Until a more systematic process is established, behavioural addictions researchers should think carefully when borrowing criteria or items from other addictive disorders. Ideally, researchers should endeavour to perform exploratory research in the first instance. This would offer more clarity in regards to which symptoms are relevant to the addictive disorder in question. Furthermore, an exploratory approach would provide opportunities for the identification of unique symptoms, which should improve construct validity.
